# Supplementary material for: Observation of nonlinear disclination states
Source: Light Sci Appl. 2023 Aug 10;12:194. doi: 10.1038/s41377-023-01235-x (PMC10412544; doi:10.1038/s41377-023-01235-x)
Supplement: Supplementary file 1 — Supplemental Materials of Observation of nonlinear disclination states [file 41377_2023_1235_MOESM1_ESM.pdf]

Supplemental Materials *of* Observation of nonlinear disclination states

Boquan Ren,[1,*] Antonina A. Arkhipova,[2,3,*] Yiqi Zhang,[1,†] Yaroslav V. Kartashov,[2,‡] Hongguang Wang,[1] Sergei A. Zhuravitskii,[2,4] Nikolay N. Skryabin,[2,4] Ivan V. Dyakonov,[4] Alexander A. Kalinkin,[2,4] Sergei P. Kulik,[4] Victor O. Kompanets,[2] Sergey V. Chekalin,[2] and Victor N. Zadkov[2,3]

[1]*Key Laboratory of Physical Electronics and Devices, Ministry of Education, School of Electronic Science and Engineering, Xi'an Jiaotong University, Xi'an, 710049, China*
[2]*Institute of Spectroscopy, Russian Academy of Sciences, Troitsk, Moscow, 108840, Russia*
[3]*Faculty of Physics, Higher School of Economics, Moscow, 105066, Russia*
[4]*Quantum Technology Centre, Faculty of Physics, M. V. Lomonosov Moscow State University, Moscow, 119991, Russia*

* The two authors constribute equally to this work.
† zhangyiqi@xjtu.edu.cn
‡ yaroslav.kartashov@icfo.eu

I. DISCLINATION LATTICES WITH DIFFERENT KEKULÉ DISTORTION COEFFICIENTS

In Fig. S1, we display the examples of disclination arrays that are controlled by the Kekulé distortion coefficient r. For $r = 0$ which is an extreme case, the six waveguides in each unit cell completely overlap with each other and locate in the middle of each unit cell. The disclination arrays in the trivial insulator phase ($r = 0.8$) and the topological insulator phase ($r = 1.68$) are also shown. In other extreme case, when $r \to \infty$, the two waveguides from neighboring cells that were initially located at different sides of the cell edge merge into one waveguide located exactly in the middle of the border between two cells. Note that the waveguides at the outer edge of the structure and at the disclination core do not have partners with which they can overlap, and on this reason their depth is smaller than depth of overlapping waveguides in this extreme case. From the perspective of the Wannier center analysis, the Wannier centers do not exit on such edges. We take the two extreme cases for qualitative analysis of trivial insulator phase and topological insulator phase, respectively. The spectral charge of the unit cell is always 3 if $r = 0$. If $r \to \infty$, the spectral charge of the corner unit cells is $3/2$, the spectral charge of the edge unit cells is 2, and the spectral charge of the unit cells around the disclination core is $5/2$.

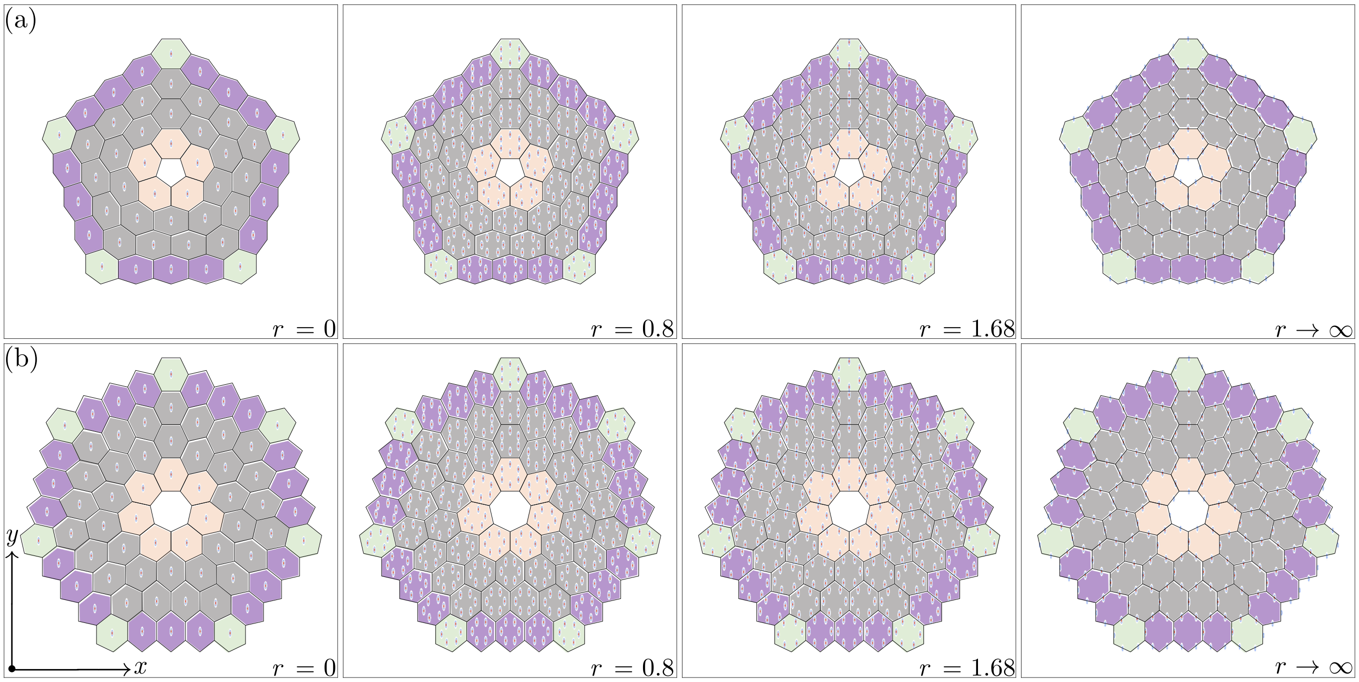

FIG. S1. Large-scale disclination arrays for different values of the Kekulé distortion coefficient r. There are 300 waveguides in the array with pentagonal core, and 420 waveguides in the array with heptagonal core. Unit cells are indicated by black hexagons with different colours of the background corresponding to the location of the cell. In the corner, edge, disclination and bulk regions, the hexagons are filled with green, purple, orange and gray colors, respectively.

II. SPECTRUM AND STATES OF THE LARGE-SCALE ARRAY

We display the linear spectra of large-scale disclination arrays with a pentagonal core in Fig. S2(a) and heptagonal core in Fig. S2(b) for $r = 1.68$. These spectra can be compared with linear spectra from the main text, presented in Fig. S1. The disclination states that are localized at the inner disclination core are indicated by the red dots. The corner-I states, corner-II states and edge states which appear at the outer boundary of the structure are also highlighted in different colors (see legends). It should be stressed that independently of the size of the structure, the number of disclination states is always five for pentagonal array and seven for heptagonal array, i.e. it is dictated by the symmetry of these structures. The same conclusion holds also for the number of corner-II states.

Representative intensity distributions of different disclination states corresponding to spectra in Figs. S2(a) and S2(b) are shown in Fig. S3(a). The propagation constant for each disclination state is also given at the bottom of each panel. It should be stressed that in this continuous system, the propagation constants of different disclination states may be close, but in general they are not degenerate. One of the reasons behind this is that our waveguides are

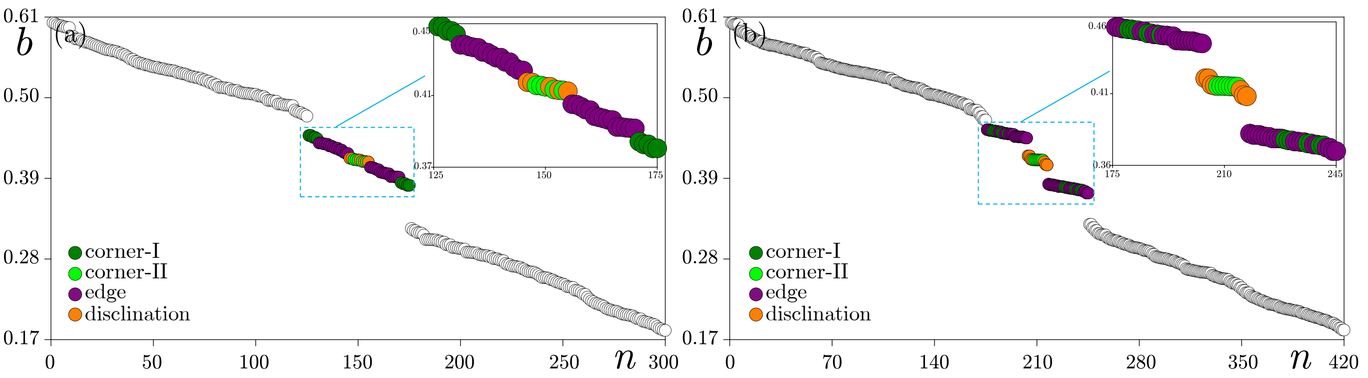

FIG. S2. Linear spectra of large-scale disclination arrays with pentagonal core (a) and heptagonal core (b). The Kekulé distortion coefficient $r = 1.68$ and potential depth $p = 5.0$.

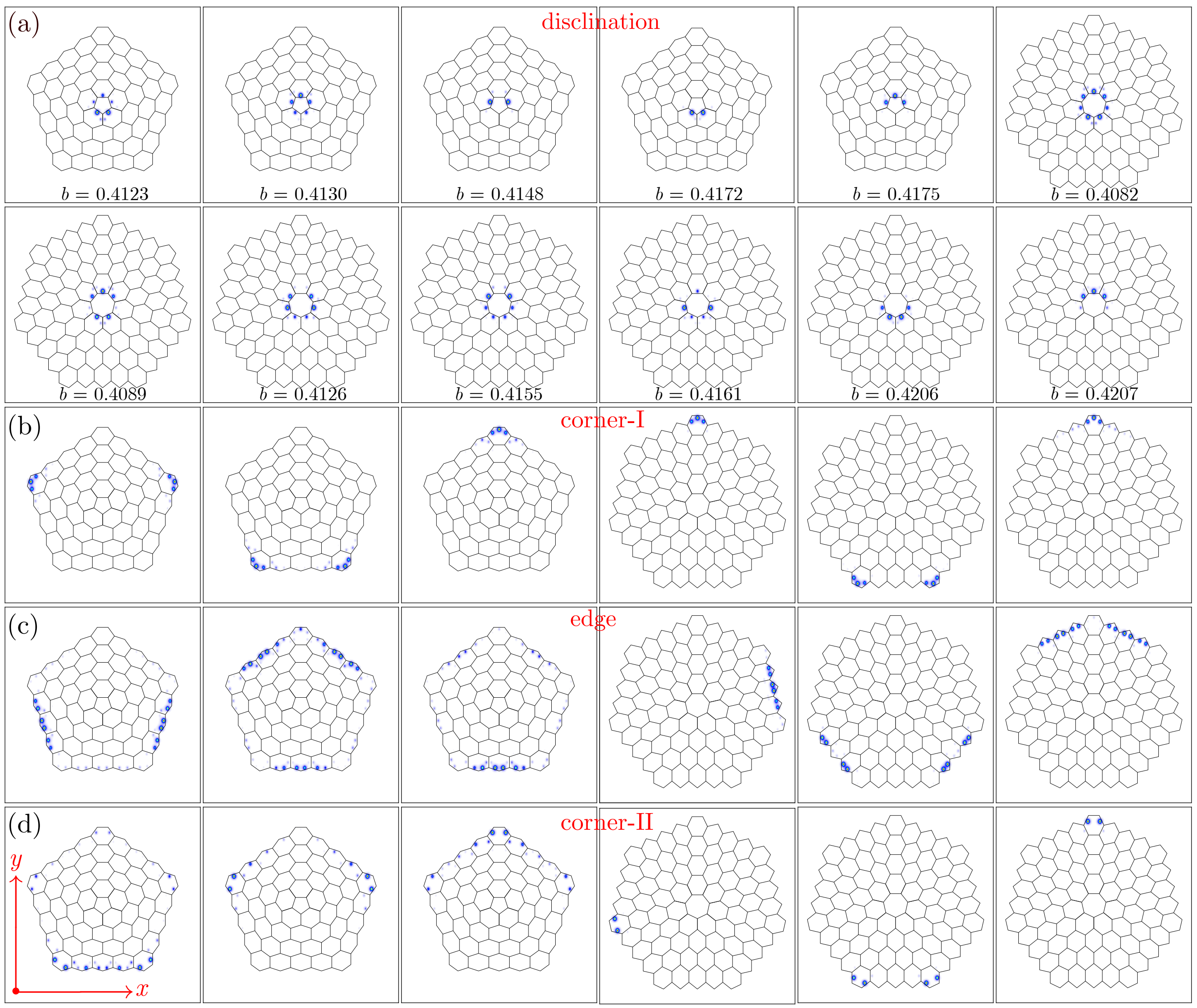

FIG. S3. Intensity distributions for the states in the gap corresponding to the spectra in Fig. S2.

elliptical with longer axis of the ellipse always parallel to the y axis. On this reason, these structures lack exact $\mathcal{C}_5$ or $\mathcal{C}_7$ discrete rotation symmetries. The small difference of the propagation constants of the disclination states explains slow beating between them that is observed in purely linear regime (in theoretical modeling of propagation over long distance z) when only one waveguide at the disclination core is excited. The examples of corner-I states, edge states and corner-II states are displayed in Figs. S3(b), S3(c) and S3(d), respectively.

III. DIFFRACTION PATTERNS IN ARRAYS WRITTEN AT DIFFERENT DEPTHS

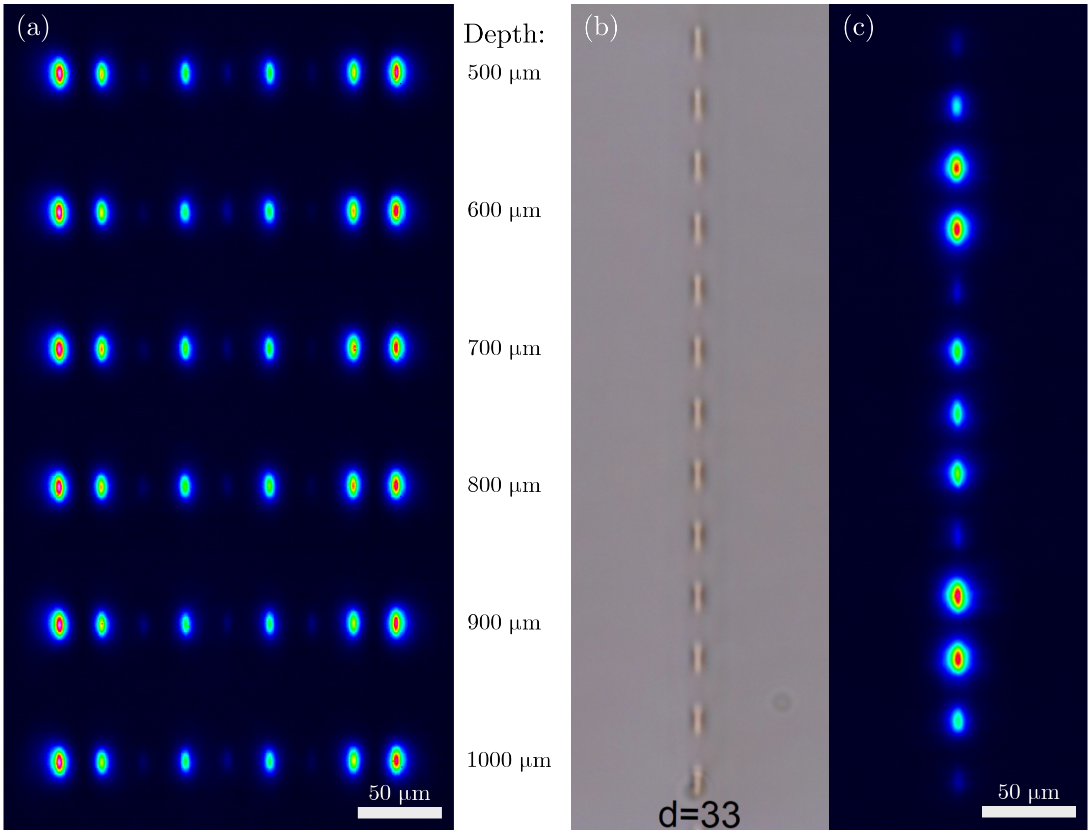

FIG. S4. Diffraction patterns upon excitation of the central waveguide in the 1D horizontal arrays with $N = 9$ waveguides, period $d = 25\,\mu$m and length 30 mm written at different depths $500 \sim 1000\,\mu$m under the surface of the sample (a). Microphotograph of the fs-laser written test 1D vertical array of $N = 13$ waveguides with period $d = 33\,\mu$m and length 50 mm (b) and diffraction pattern in such array observed upon excitation of the central waveguide (c).

The waveguide arrays shown in Figs. 1(a,d) from the main text were inscribed using an aspheric lens with low NA $= 0.3$, where optical aberrations contribute negligibly near the optimal depth and no additional aberration correction is used. The optimal depth was selected after inscribing the series of test 1D horizontal arrays of $N = 9$ waveguides with the period of $d = 25\,\mu$m and length 30 mm at different depths $100 \sim 2000\,\mu$m under the surface of the sample. The most similar diffraction patterns upon excitation of the central waveguide were observed at the depth range of $500 \sim 1100\,\mu$m [see Fig. S4(a)], so that the depth of $800\,\mu$m was chosen as the optimal one. It should be stressed that the shapes of the waveguides are almost identical at this depth range [see Fig. S4(b)]. Notice also that the diffraction pattern observed upon excitation of the central waveguide of the test 1D vertical array with $N = 13$ waveguides, period $d = 33\,\mu$m and length 50 mm [see Fig. S4(c)] is also highly symmetric. This clearly shows that this writing technology allows to create large-scale arrays with practically identical waveguides.

IV. NONLINEAR DISCLINATION STATES FAMILY IN DEFOCUSING NONLINEAR MEDIA

To stress universality of nonlinear disclination states we show here that they can emerge even in arrays with defocusing nonlinearity. The Schrödinger-like equation for the dimensionless light field amplitude ψ describing light propagation in such array can be written as

$$i\frac{\partial\psi}{\partial z} = -\frac{1}{2}\left(\frac{\partial^2}{\partial x^2}+\frac{\partial^2}{\partial y^2}\right)\psi - \mathcal{R}(x,y)\psi + |\psi|^2\psi. \tag{S1}$$

The family of soliton solutions of Eq. (S1) bifurcating from the linear disclination states can be obtained using the Newton method. Corresponding dependencies of power U on propagation constant b and examples of profiles of nonlinear disclination states are presented in Fig. S5 for the arrays with pentagonal and heptagonal disclination cores. Importantly, because the sign of nonlinearity is different now, the bifurcation from linear disclination states occurs now in the direction of decreasing b, towards lower edge of the gap. Except for this, solitons in defocusing medium show very similar behaviour to states discussed in the main text—they are also stable and their localization depends on the position of propagation constant in the gap. For instance, when the propagation constant shifts into the allowed gray band, the solitons exhibit delocalization.

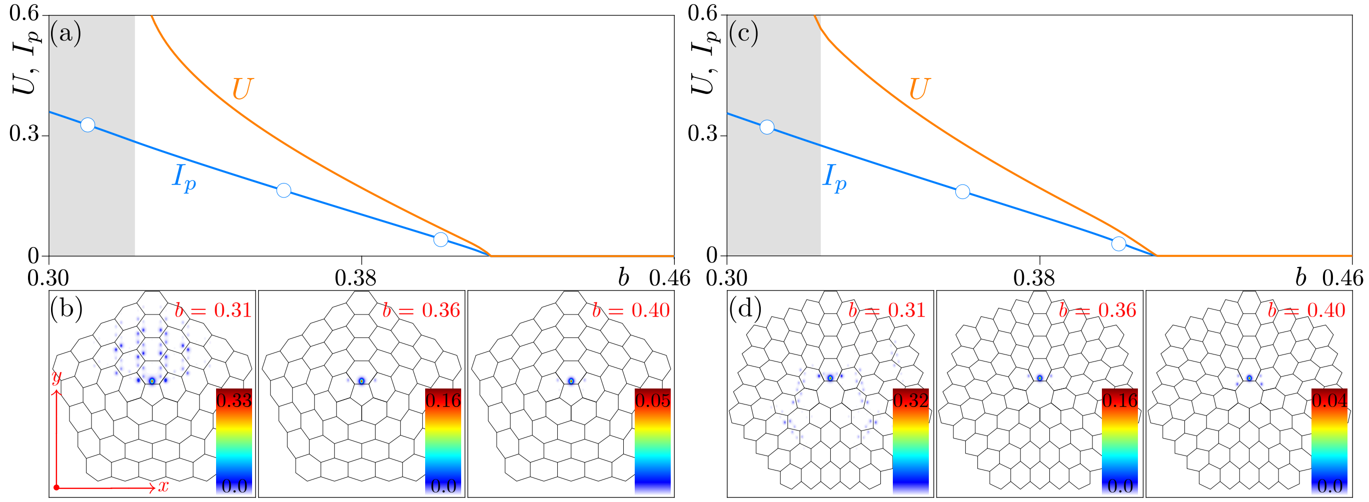

FIG. S5. (a) Peak intensity I_p (blue solid curve) and power U (orange solid curve) of the nonlinear disclination states vs propagation constant b in the array with the pentagonal core. Gray regions represent the bulk band, while the vertical dotted color lines show propagation constants of linear corner-I, edge, corner-II, and disclination states. (b) Intensity distributions of selected nonlinear disclination states with different propagation constants that correspond to circles in (a). (c,d) The families of nonlinear disclination states and examples of their profiles in the array with heptagonal core. Intensity distributions in (b) are shown within the window $-40 \le x, y \le 40$, while those in (d) are shown within the window $-46 \le x, y \le 46$. In both cases distortion parameter $r = 1.68$.

V. NONLINEAR DISCLINATION STATES IN SATURABLE MEDIA

Nonlinear disclination states can also be obtained in materials with other types of nonlinearity beyond cubic Kerr nonlinearity. For example, photorefractive materials are characterized by saturable nonlinear response. Light propagation dynamics in photorefractive material (such as SBN) with saturable nonlinearity and optically induced disclination lattice can be described by the following equation [1, 2]

$$i\frac{\partial\psi}{\partial z} = -\frac{1}{2}\left(\frac{\partial^2}{\partial x^2}+\frac{\partial^2}{\partial y^2}\right)\psi + \frac{E}{1+I(x,y)+|\psi(x,y,z)|^2}\psi, \tag{S2}$$

where I is the intensity of the lattice-creating beam scaled to the background illumination intensity $I_{\rm bg}$, and $E = (1/2)k_0^2 r_0^2 n_e^4 \gamma_{33} E_0$ is the scaled bias field. The typical parameters [3] are: the wavelength $\lambda_0 = 632.8\,{\rm nm}$, $k_0 = 2\pi/\lambda_0$, the unperturbed refractive index $n_e = 2.2817$, the bias field $E_0 = 6.82 \times 10^4\,{\rm V/m}$, and the involved electro-optic coefficient $\gamma_{33} = 250\,{\rm pm/V}$. In Eq. (S2), the function I is written as $I(x,y) = |\mathcal{R}|^2$, where $\mathcal{R}$ has the same form

as lattice considered in the main text, except that here we take $p = 3$ that corresponds to an average intensity of $I_{av} \approx 11.4\,\mathrm{mW/cm}^2$. In Fig. S6, we show the spectrum [Fig. S6(a)] of the disclination lattice with a pentagonal core induced in the SBN crystal [Fig. S6(b)]. The five disclination states are shown in Fig. S6(c). One finds that details of the spectrum for these parameters are quite similar to spectra presented in the main text [Figs. 1(a)-1(c)]. We also obtained the family of nonlinear disclination states bifurcating from linear disclination modes, that is presented in Fig. S6(d). Representative profiles of solitons on disclination core are shown in Fig. S6(e). Just like in cubic medium, such states are well localized inside the gap and exhibit delocalization when their propagation constant shifts into the band.

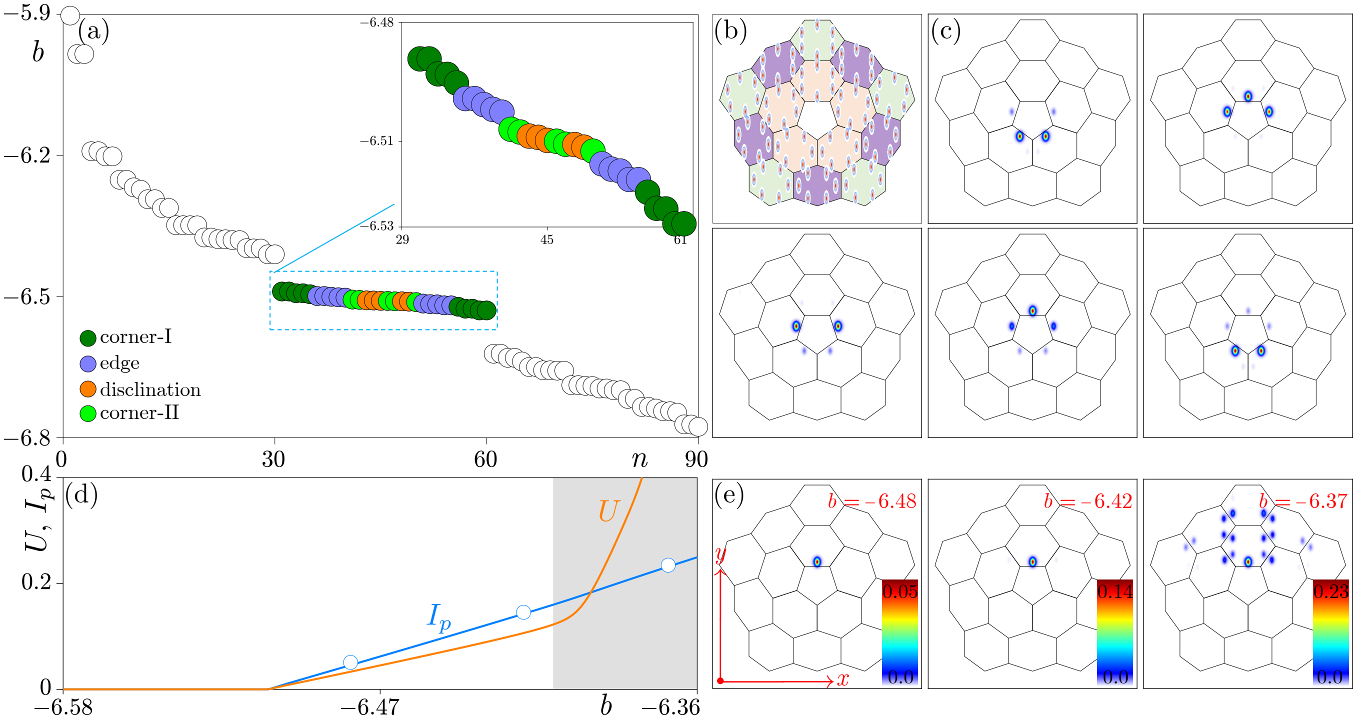

FIG. S6. (a) Spectrum of the pentagonal disclination lattice with $r = 1.68$ induced in the SBN crystal that is shown in (b). (c) Intensity profiles of the five disclination states. (d) Nonlinear disclination state family bifurcating from the linear counterpart. (e) Intensity profiles of representative nonlinear disclination states. Panels in (b), (c) and (e) are shown in the window $-18 \le x, y \le 18$.

Note that hot atomic vapors [4] also represent materials with saturable nonlinear response, where topological structures can be created, and that they have been widely adopted as an ideal platform in nonlinear topological photonics.

VI. LASING IN DISCLINATION STATES

Finally, we briefly discuss here the possibility of lasing in the disclination states. To realize such a lasing we consider the dissipative disclination array imprinted in the material with nonlinear absorption, where gain is provided only on the sites belonging to the disclination core to stimulate preferential amplification of the disclination states. Light propagation dynamics in such a structure is described by the dissipative version of the nonlinear Schrödinger-like equation that in the dimensionless units reads as

$$i\frac{\partial\psi}{\partial z} = -\frac{1}{2}\left(\frac{\partial^2}{\partial x^2} + \frac{\partial^2}{\partial y^2}\right)\psi - (\mathcal{R} - i\mathcal{I} + i\gamma)\,\psi - (1 + i\alpha)|\psi|^2\psi, \tag{S3}$$

where $\mathcal{I}$ is the spatially inhomogeneous gain that is nonzero only for sites on the disclination core of the array $\mathcal{R}$, γ is the coefficient of linear losses that are assumed to be uniform, α is the coefficient characterizing nonlinear losses stemming from all sources, including intrinsic nonlinear losses of the medium and gain saturation in the first

approximation. The inhomogeneous gain landscape in Eq. (S3) is described by the function

$$\mathcal{I}(x,y) = p_{\rm im} \sum_c e^{-(x-x_c)^2/a_x^2-(y-y_c)^2/a_y^2},$$

where x_c, y_c are the coordinates of the corresponding waveguides belonging to disclination core and $p_{\rm im}$ is the normalized gain amplitude. Here, we consider the structure with pentagonal core as an example.

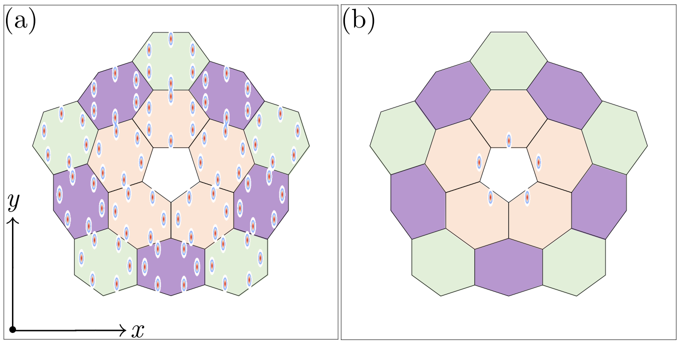

FIG. S7. (a) Profile of disclination lattice with pentagonal core $\mathcal{R}$. (b) Gain landscape $\mathcal{I}$. In both cases the window shown is $-26 \leq x, y \leq 26$.

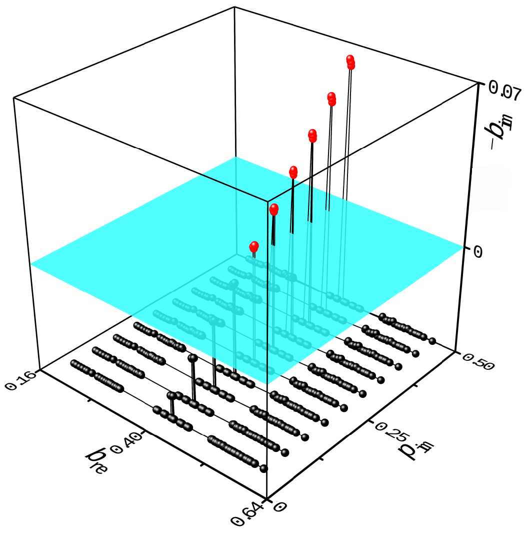

FIG. S8. Transformation of linear spectrum of the system with increase of gain amplitude $p_{\rm im}$. Disclination states exhibit amplification when imaginary part of their propagation constant $-b_{\rm im} > 0$ (i.e. when it crosses blue plane) around $p_{\rm im} \sim 0.25$. The red dots correspond to amplified disclination states, while states corresponding to black dots experience damping.

Corresponding lattice structure and gain landscape are presented in Figs. S7(a) and S7(b). First of all, we calculate linear spectrum of the array with inhomogeneous gain and uniform losses $\gamma = 0.05$ by neglecting the nonlinear term and nonlinear absorption in Eq. (S3), and the result is shown in Fig. S8, where we plot imaginary part of the eigenvalue $-b_{\rm im}$ of the eigenmodes as a function of real part $b_{\rm re}$ and gain amplitude $p_{\rm im}$. The modes exhibit amplification only when $-b_{\rm im} > 0$ (otherwise they are attenuated), and this amplification naturally occurs first for disclination states when gain coefficient exceeds the threshold value $p_{\rm im} \sim 0.25$, which is an indication of possibility of lasing in such modes. Next we take into account nonlinear terms and nonlinear absorption in Eq. (S3) and obtain *stationary* lasing disclination states using the Newton method (for such states the growth of amplitude is eventually arrested by nonlinear absorption, so that the system reaches the stage where stable dissipative state forms, whose amplitude does not change with z). In Fig. S9(a) and S9(b) we show peak amplitude $A = |\psi|_{\rm max}$ and propagation constant b of the lasing disclination state as a function of $p_{\rm im}$ for different coefficients of nonlinear absorption α. One can see that disclination states emerge above lasing threshold $p_{\rm im} \sim 0.25$ and their amplitude monotonically increases

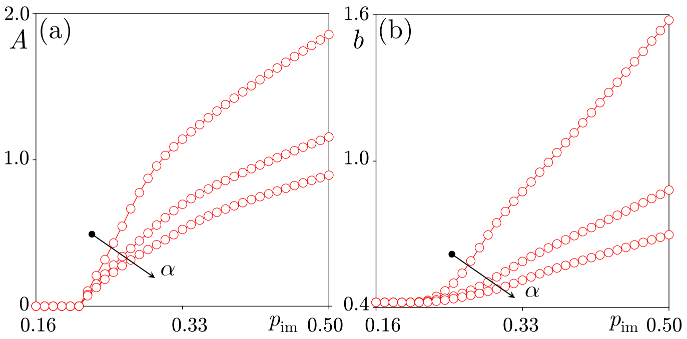

FIG. S9. The dependence of peak amplitude A (a) and propagation constant b (b) of the lasing state on gain amplitude $p_{\rm im}$ for different values of nonlinear absorption coefficient $\alpha = 0.1$, 0.2 and 0.3 increasing in the direction shown by the arrow.

with increase of gain amplitude $p_{\rm im}$. For fixed gain amplitude $p_{\rm im}$ the amplitude of such states decreases with increase of nonlinear absorption α.

---

[1] Z. Hu, D. Bongiovanni, D. Jukić, E. Jajtić, S. Xia, D. Song, J. Xu, R. Morandotti, H. Buljan, and Z. Chen, "Nonlinear control of photonic higher-order topological bound states in the continuum," Light Sci. Appl. **10**, 164 (2021).

[2] H. Zhong, S. Xia, Y. Zhang, Y. Li, D. Song, C. Liu, and Z. Chen, "Nonlinear topological valley Hall edge states arising from type-II Dirac cones," Adv. Photon. **3**, 056001 (2021).

[3] Q. Fu, P. Wang, C. Huang, Y. V. Kartashov, L. Torner, V. V. Konotop, and F. Ye, "Optical soliton formation controlled by angle twisting in photonic moiré lattices," Nat. Photon. **14**, 663 (2020).

[4] Z. Y. Zhang, R. Wang, Y. Q. Zhang, Y. V. Kartashov, F. Li, H. Zhong, H. Guan, K. Gao, F. L. Li, Y. P. Zhang, and M. Xiao, "Observation of edge solitons in photonic graphene," Nat. Commun. **11**, 1902 (2020).
